# The Effects of Thermal Water Physical Exercise in Patients with Lower Limb Chronic Venous Insufficiency Monitored by Bioimpedance Analysis

**DOI:** 10.3390/diagnostics10110889

**Published:** 2020-10-31

**Authors:** Erica Menegatti, Anselmo Pagani, Giampiero Avruscio, Marianna Mucignat, Sergio Gianesini

**Affiliations:** 1 Department of Morphology, Surgery and Experimental Medicine, Vascular Diseases Center, University of Ferrara, 44124 Ferrara, Italy; anselmo.pagani@edu.unife.it (A.P.); marianna.mucignat@unife.it (M.M.); gnssrg@unife.it (S.G.); 2 Department of Cardiac, Thoracic and Vascular Sciences, Angiology Unit, Padova University Hospital, 35028 Padua, Italy; giampiero.avruscio@aopd.veneto.it; 3 Vascular Surgery Residency Program, University of Ferrara, 44124 Ferrara, Italy; 4 Department of Surgery Uniformed Services University of the Health Sciences, Bethesda, MD 20814, USA

**Keywords:** bioimpedance, volume, edema, exercise, thermal water, chronic venous insufficiency

## Abstract

Background: Lower limb chronic venous diseases (CVD) lead to possible edema. The aim of the present investigation was to study the effect of thermal aquatic standardize exercise on lower limb edema control in CVD patients assessed by bioimpedance analysis (BIA). Methods: Seventeen patients (34 legs) affected by CVD clinical class CEAP C3, 4c, Ep, As, Pr were included. All the cohort performed a standardized exercise protocol in thermal water environment for a total of five sessions. BIA, leg volume, and heart rate at rest were measured. Results: After the five exercise sessions, BIA showed a significant percentage of extracellular water (ECW) reduction from 42.1 ± 5.8 to 41.24 ± 5.5%; *p* < 0.001. Moreover, an improvement of resistance (*p* < 0.0009) and reactance (*p* < 0.009) was assessed. At the same time, the leg volume reduction rate was 15.7%, *p* <0.0001. A moderate-strong correlation was found between % ECW and leg volume variation (R = 0.59, *p* < 0.01). Finally, a significant HR at rest reduction was recorded, *p* < 0.0001. Conclusion: The investigated exercise protocol significantly affects the lower limb volume, and BIA parameters related to the tissue drainage improvement. The correlation founded between the ECW rate and volume variations suggest the possible use of BIA as a biomarker for monitoring the treatments aimed to reduce edema in CVD.

## 1. Introduction

Lower limb chronic venous disease (CVD) is a pathological condition which affects a large part of the population and leads to venous blood pooling and related edema formation [1].

In the initial CVD stages, the lymphatic and venous systems balance each other, while, in advanced stages, the lymphatic drainage is unable to compensate for the excessive venous filtration, thus leading to edema development [2,3].

Bioimpedance analysis (BIA) is a technique capable of measuring the impedance of the human body, and it is funded on the ability of biological tissue to impede electric current the so-called resistance [4,5].

There are different BIA measurement approaches: Single frequency, multiple frequencies, and broadband frequency spectrum signals also known as bioimpedance spectroscopy (BIS). Moreover, BIA can be differentiated in its use across the whole body, through body segments, or other alternative analysis methods such as vector bioimpedance analysis and real time bioimpedance methods [4].

BIA presents the advantages of non-invasiveness, safety, non-operator dependency, portability, and cost-effectiveness. For all these advantages, several studies have been conducted on BIA and its applications in body composition measurements and evaluation of clinical conditions [4]. Nevertheless, the limited BIA accuracy in assessing body composition has been reported, in particular in pathological fluids accumulation and in altered body geometry [6,7].

Body fluids are represented by the volume of fluids inside a human body. Total body water (TBW) is made by intracellular water (ICW) (fluid inside the cellular mass) and extracellular water (ECW) (fluid outside the cells) [4,8].

The body fluid composition assessment made by BIA is based on the inverse correlation between body resistance and the TBW, by means of the calculation on different prediction equations [9,10]. Moreover, other important physical parameters of BIA are resistance (R), indicating the fluid contents, and reactance (Xc), representing tissue composition [11,12,13]. Phase angle (PA) is another relevant BIA parameter, obtained by the relationship between R and Xc, and thus associated with cellularity, cell size, and integrity of the cell membrane. Low phase-angle values have been reported in case of cellular death or altered permeability of the membranes following their integrity loss. PA represents an important index for monitoring the presence and evolution of chronic inflammatory processes [14,15].

BIA use for lymphedema investigation was first reported in the early 1990s, considering ECW evaluation as a fundamental parameter in the assessment of venous-lymphatic drainage alteration [16]. There have been several studies comparing BIA, ultrasonography, and limb circumference measurement for early diagnosis of lymphedema, but they were mainly limited to the upper limb [17,18,19,20]. On the contrary, only a few investigations correlated these outcomes in chronic lower limb lymphedema [13,21].

Exercise prescription, especially in an aquatic environment, has been demonstrated to be an effective adjuvant therapy for patients affected by lower limb lymphedema [22,23,24,25,26,27]. The aim of the present investigation was to study the effect of thermal aquatic standardized exercise on lower limb edema control in phlebolymphedema patients assessed by BIA.

## 2. Methods

### 2.1. Study Design and Population

This is a prospective study including 34 legs of 17 patients (12F/5M, mean age 61 ± 11; body mass index (BMI) 27 ± 5; venous clinical severity score VCSS 9 ± 3) affected by CVD (CEAP C3, 4c, Ep, As, Pr) [1].

Inclusion criteria were:

- Age from 18 to 80 years old;
- BMI < 35;
- CVD with visible varicose veins and edema.

Exclusion criteria were:

- Cardiac comorbidity (e.g., congestive heart failure, cardiomyopathy, coronary artery disease);
- lower limb arterial disease;
- severe biochemical alterations (e.g., diabetes mellitus, hypothyroidism);
- chronic kidney disease;
- fear of water;
- venous active drugs and/or graduated compression use;
- use of drugs potentially leading to edema.

At the study enrollment, all the patients underwent a detailed ultrasound Doppler (Esaote MyLab25, Genoa Italy) scanning of the deep, superficial, and perforating vein system, together with a vascular specialist visit. The use of graduated compression stockings during the investigation time was excluded, since it constitutes a potential bias.

### 2.2. Exercise Program

All the cohort underwent a standardized exercise protocol [27] in a thermal water environment designed to mobilize all the joints and empowering the muscular kinetic chains of the lower limb. Each exercise session was 30 min long, for a total of 5 sessions in 14 days.

The standardized physical activity protocol was composed by the following exercises:

- Warming up by a cycling-like activity, in a supine position while holding the handrail;
- tip–toe exercise (four series of ten repetitions each);
- hip flexion–extension on the non-weight bearing limb (four series of ten repetitions each);
- tip–toe exercise on a step (four series of ten repetitions each);
- knee flexion–extension (four series of ten repetitions each);
- forward and backward walking for 5 min;
- lateral walking on both sides for 5 min;
- cycling-like single push while standing up on the contralateral limb (four series of ten repetitions each);
- ankle flexion–extension while keeping the knee bent at 90° (four series of 10 repetitions each);
- cool-down by a cycling-like activity, in a supine position while holding the handrail.

All the exercises were performed in a thermal salso-bromo-iodine water pool of an authorized thermal center of Abano Terme (PD)—Italy. The water of the Abano and Montegrotto (Veneto Region, Italy) Euganean hills thermal basin reaches 80/90 °C before arriving at the surface, and the characteristics of the thermal water is summarized in Table 1 [28].

The water temperature was maintained at 33 °C and the pool depth was 120 cm with an area of 25 m^2^.

### 2.3. Bioimpedance Analysis (BIA)

Before starting the data collection, all the patients were trained to carry out the BIA measurements by themselves by means of a portable multifrequency instrument (Biody Xpert IITM; eBIODY SAS La Ciotat, France). The multiple frequencies delivered by the instrument ranged from 5 kHz to 200 kHz, while R, Xc, and PA were settled at 50 kHZ [11,12,13,14,15]. Soon after the recording, all the data were transferred via Bluetooth technology on a computer and stored under a personal patient folder.

BIA was performed before the exercise session at baseline (T0), at 7 days (T7), and at 14 days (T14), respectively.

The following BIA parameters have been assessed:

- ECW % calculated according to the following formula: (extracellular water/extracellular + intracellular water) × 100 [29].
- R as the expression of extracellular fluids amount. This latter is inversely proportional to the extracellular fluids components [13].
- Xc depends on the interaction between cell membranes and BIA current, and it is directly proportional to cell density [13].
- PA is obtained through the relationship between direct measures of resistance (R) and reactance (Xc). In young healthy subjects, the value ranged from 6° and 7°. In general, values < 5° indicate a rupture of cell membranes or an accumulation of extracellular fluids, and values around 10° indicate severe dehydration [14,15].

### 2.4. Lower Limb Volumetry

Before and after every single exercise session, the lower limb volume was assessed by water displacement leg volumetry (WP), which is the gold standard for volume assessment, and it is a highly reproducible method based on a simple physical principle [30].

The patient is sitting down in front of the WP, which is completely filled of water (13 L), and is asked to put each leg inside the water displacement. The volume that overflows in the adjacent container following the introduction of the leg represents an indicative parameter of limb volume.

The overflowing water container is calibrated on a graduated scale reporting every 50-mL variation.

The measurement of both legs was performed following the correct standard reported in the literature [30,31]:

- The water level was above the pretibial region;
- the water temperature ranged from 28 °C to 32 °C;
- the subjects were measured in sitting and resting position
- the volume assessment was performed right before and right after the 5 exercise sessions
- the time of the measurements was between 9 and 12 a.m.

### 2.5. Heart Rate at Rest

The heart rate was measured at rest (resting-HR) by a portable pulse oximeter (JUMPER Finger 500A Meter Pulse Portable, Germany), at baseline, and after 2 days from the end of the exercise program.

All subjects gave their informed consent for inclusion before they participated in the study.

The study was conducted in accordance with the Declaration of Helsinki, and the protocol was approved by the Ethics Committee of Ferrara University–Hospital [approval number: EM83-2019_UniFe/170293_EM1–23/01/2019].

### 2.6. Statistical Analysis

Prism 8—vers.8.2.1, 2019 (GraphPad Softwere Inc. San Diego, CA 92108, USA) was used for statistical analysis. The data were expressed as mean and standard deviation or percentage. The Kolmogorov-Smirnov test was used to assess the data distribution. Clinical data relating to the two legs of each patient were averaged to obtain a single numerical value to simplify the analysis and the amount of data to present.

The differences in ECW, R, Xc, PA, lower limb volume, and resting-HR were performed using the two-tailed Student t-test for paired data or Wilcoxon test as appropriate.

Pearson’s correlation coefficient was used to calculate the linear correlation between ECW variation (T0–T14) and leg volume variation (T0–T14). The R cut-off points were the following: 0.00–0.39 “weak correlation”; 0.40–0.89 “moderate-strong correlation”; 0.90–1 “very strong correlation” [32]. Statistical significance was defined as *p* < 0.05.

## 3. Results

All the included participants accomplished the protocol and neither major nor minor adverse events were reported.

### 3.1. BIA

**Figure 1:**
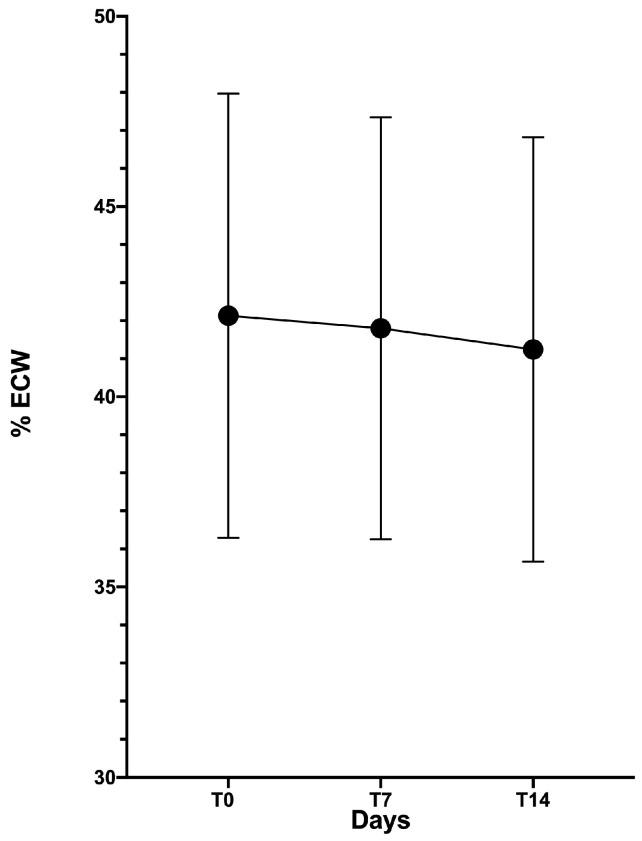
Decreasing in extracellular water rate (% ECW) from baseline (T0); to 7 days (T7) and 14 days (T14).

BIA showed a progressive significant % ECW reduction both during the first week (T0–T7) and the second week (T7–T14) of exercise program, Figure 1.

**Figure 2:**
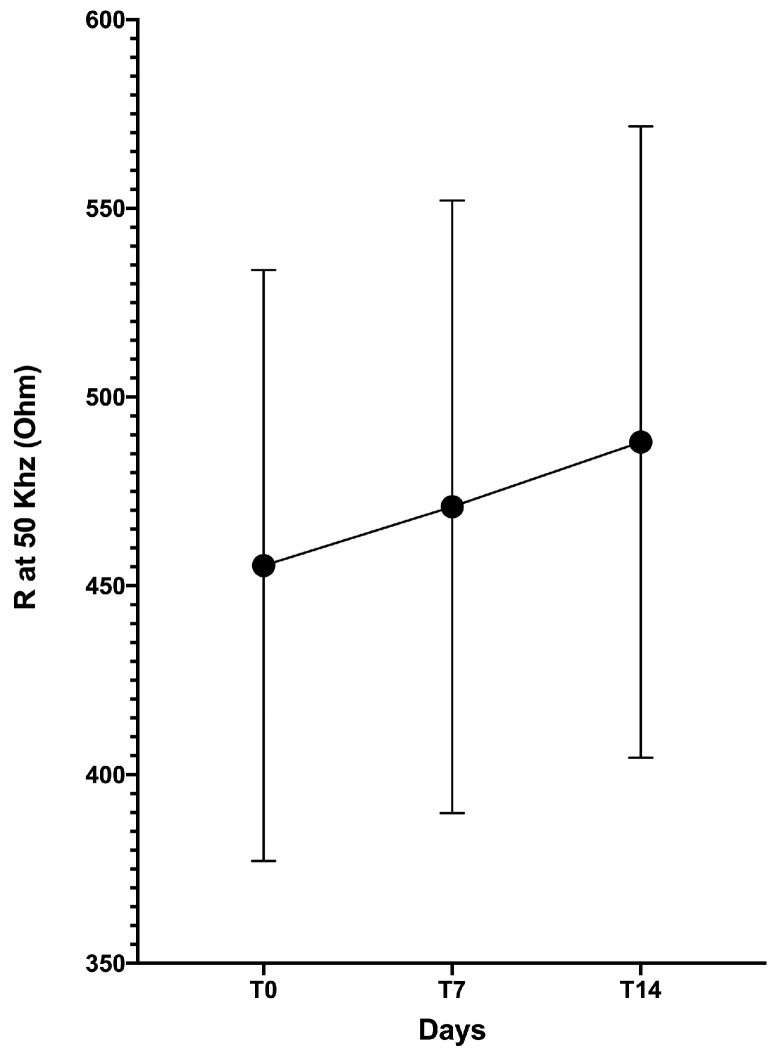
Increase in resistance (R) from baseline (T0); to 7 days (T7) and 14 days (T14).

A statistically significant improvement of R was recorded after 5 exercise sessions (Figure 2).

Regarding the Xc, no significant differences were assessed in the first week (T0–T7), whereas a significant improvement was recorded during the second week of the exercise program (T7–T14), leading to a significant increasing of such a parameter from baseline (T0) to the end of the protocol (T14).

Finally, no statistically significant differences were measured among the measured PA.

Table 2 shows the detailed variations of BIA parameters.

### 3.2. Lower Limb Volume

**Figure 3:**
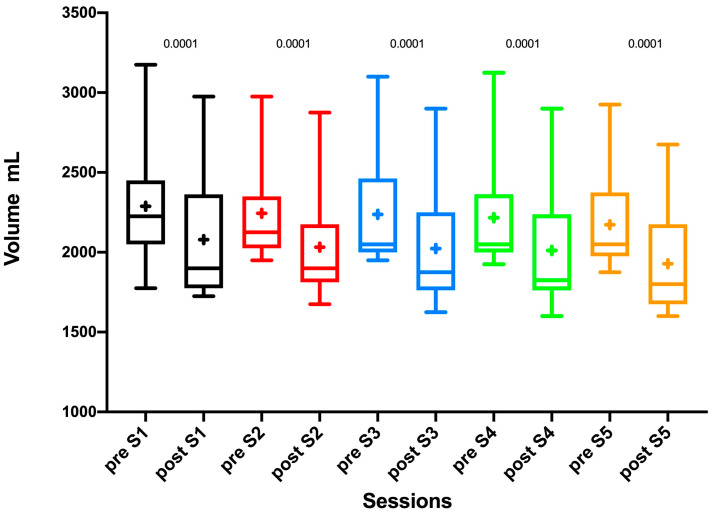
Box and whiskers of leg volume variation before (pre) and after (post) the five exercise sessions (S1, S2, S3, S4, S5). The symbol **+** indicates the mean.

A statistically significant volume reduction was measured along the five sessions (Figure 3), and the detailed values are shown in Table 3. The volume variation rate from T0 to T14 was 15.7%, *p* <0.0001.

**Table 3:** Leg volume variation pre- and post-exercise of every single session.

Session	Leg Volume Pre-Exercise (mL)	Leg Volume Post-Exercise (mL)	Variation (%)	*p*
**S1**	2288 ± 348	2079 ± 385	−9.1%	0.0001
**S2**	2244 ± 310	2032 ± 338	−9.4%	0.0001
**S3**	2237 ± 327	2024 ± 356	−9.5%	0.0001
**S4**	2216 ± 335	2012 ± 367	−9.2%	0.0001
**S5**	2172 ± 310	1928 ± 321	−11.2%	0.0001

**Figure 4:**
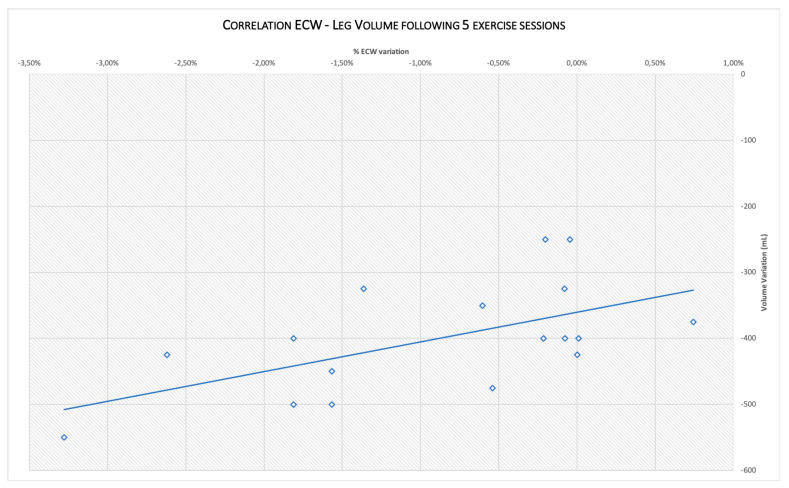
Correlation between leg volume and % ECW variation from baseline (T0) to the end of the exercise program (T14).

Furthermore, a “moderate–strong” correlation was found among the assessed % ECW and leg volume variation (Pearson coefficient R = 0.59, *p* < 0.01), see Figure 4.

### 3.3. Resting-HR Measurement

**Figure 5:**
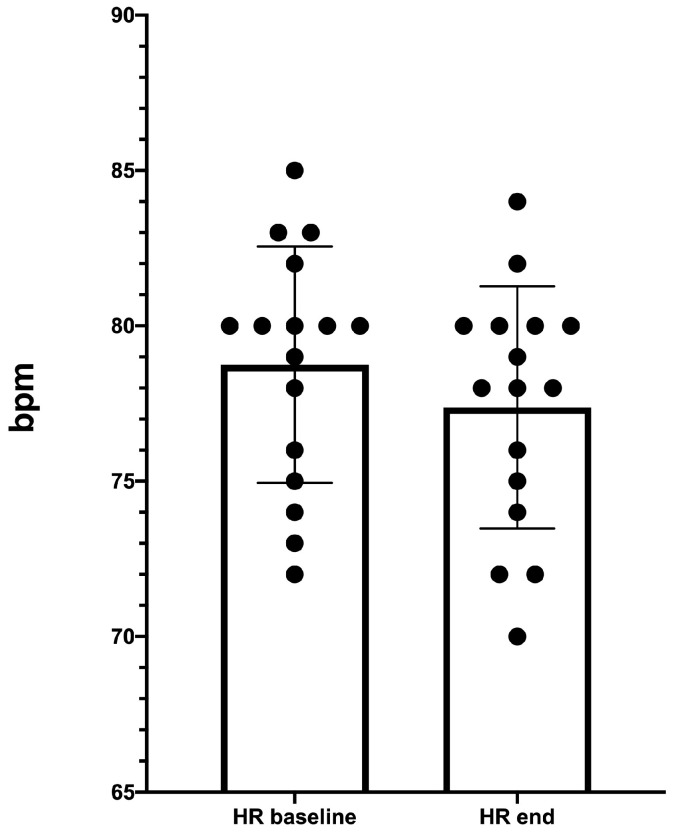
Heart rate (HR) at rest decreasing after the five exercise sessions. HR baseline = T0; HR end = 2 days after the end of the program.

A statistically significant resting-HR decreasing was recorded following the five exercise sessions (from 78.8 ± 3.8 bpm to 77.4 ± 3.9 bpm; *p* < 0.0001), see Figure 5.

## 4. Discussion

Despite the apparently obvious beneficial pressure effect of water in case of CVD, the use of such aquatic environments for lower limb drainage improvement is currently affected by a paucity of investigations on the topic [27,33]. Moreover, the salso-bromo-iodine water has demonstrated beneficial effects on chronic inflammation of different origin with a consistent literature body, especially on its use for musculo-skeletal diseases [28,34,35,36,37,38,39,40].

The most relevant finding of our study is the significant edema reduction measured by the gold standard method (WP), and its moderate–strong correlation with the % ECW variation. For this reason, BIA become of particular interest, integrating the limb volume measurements that are not able to discriminate between intra and extracellular water. Among the BIA parameters, R and Xc have been found to be useful in the assessment of CVD-related edema.

Both of them showed a significant improvement along the exercise sessions according to the trend of ECW rate and lower limb volume. Similar to previous studies on decongestive treatments for lymphedema [13] and CVD treatments monitored by bioimpedance spectroscopy [41], the R demonstrated a higher sensitivity than Xc for edema follow-up.

On the contrary, according to this study, PA did not show significant sensitivity in the CVD-related edema monitoring. The finding could be justified by PA correlation with the cellular metabolism rather than fluid dynamics [14,15,42]. In the literature, PA was demonstrated to be reliable in the assessment of the health condition and metabolic status, thus future investigations should be addressed to measure its utility in CVD patients affected by the most severe stages and with significant comorbidities, such as obesity, diabetes, and renal or cardiovascular failures.

Our study pointed out that PA still remains within the normal range, indicating the health cellular status of our population, without variations following five thermal water exercise sessions.

The herein reported data pave the way for further detailed investigations aimed to assess the effect of different treatments associated with muscle pump activation on specific edema components, such as the extracellular water. In-depth investigations of the correlation between limb volume and fluid component variations following standardized exercise protocols and external compressions and/or venous active drugs use are needed.

Interestingly, the experimental exercise protocol was not able to decrease the lower limb volume in between the single sessions, thus suggesting the need of proper exercise/graduated compression stocking use in between.

Aquatic immersion presents significant potential benefits for lower limb CVD edema control, modulating the transmural pressure (TMP). The rationale of the therapeutic effects of the aquatic exercise is based on the physics law regarding the TMP and Stevin’s Law. [27,33]

Aquatic environment also enhances muscle activation and joint mobilization by removing part of the gravitational load, increasing proprioceptive system stimulation, amplifying the external sensorial stimulus [28,34]. All these physical effects are due to the buoyancy of the aquatic mean.

Moreover, the hydrostatic pressure exerted by water generates a naturally graduated compression from the ankle to the thigh, mimicking an enhanced graduated compression stocking, whose tolerability is maximized since it is not perceived by the patient.

Chronic venous and lymphatic disorders are characterized by increased TMP leading to the ultrafiltration of water and peptides in the interstitial compartment, which is the main mechanism of edema pathogenesis according to Starling law [43,44].

Standard treatment of advanced CVD stages complicated by edema includes surgery and compression. Surgery improves TMP by reducing the internal venous pressure whereas compression by reducing the TMP increasing the external pressure [45,46]. Conservative based treatments of CVD embrace either the ones acting though decreasing the gravitational hydrostatic pressure such as postural exercises leg elevation, or the ones working to increase the external venous pressure like bandaging/stockings, [45,46] pneumatic compression [45], and, of course, immersion into liquids [27,33].

Our aquatic exercise protocol, similar to compression, mechanically improves TMP by increasing the external pressure together with muscle pump training and correct joint mobility activation.

It has been described that methods based on TMP reduction act on the modulation of some plasma inflammatory markers, including key inflammatory molecules mediating edema as a manifestation of chronic inflammation [46]. Moreover, in CVD patients, in particular the most severe stages (C3, C4, C5) and during disease relapses, high circulating levels of the cytokine-chemokine cascade have been assessed [47].

Fluid reabsorption, together with lower limb muscle strengthening and proper ankle joint activation, are transmitted into the microcirculation system influencing chronic inflammation, potentially involving cytokines cascades, as already demonstrated in patients affected by chronic venous disease [47,48,49].

Such an effect together with the adjuvant effect of salso-bromo-iodine water might explain the reason of our results.

In addition, the robust correlation founded between WP and BIA suggest the possible use of this method as a biomarker for monitoring the treatments aimed to reduce edema in CVD.

There are several advantages including the possible use of portable instruments, the cost-effectiveness, the quick and easy operator training, and the reduced time in test performance. Thus, in perspective, this portable tool becomes helpful to avoid the time-consuming measurement protocol, especially in the out-patient setting, which is particularly interesting for any study aimed to assess the effects of exercise on the vascular system.

Water training also has some mild effects on the cardiovascular system such as peripheral vasodilation, blood pressure reduction, initial tachycardia followed by reduction of HR at rest, and an increase in cardiac workload [34,50,51]. Our preliminary results also confirm the cardiovascular adaptation with a significant reduction of resting-HR at the end of the five exercise sessions.

The major limitation of our study is the lack of a control group. However, this study was aimed to assess the possibility of using BIA as biomarker of lower limb chronic edema, as well as its ability to monitor the changes over time as compared to the gold standard.

Another shortcoming is the use of an instrument that does not allow a sectorial evaluation. R, Xc, and % ECW are calculated on the whole body and not limited to the lower limb, and for such reason the use of devices allowing a sectorial evaluation could lead to even more accurate data, but it would not have the advantages of a portable tool. Nevertheless, these parameters present a statistically significant variation, making it a potentially useful portable tool for CVD-related edema monitoring.

Moreover, a complete circulatory cytokines assessment which potentially might correlate with the measured anti-edema effect can corroborate the findings of this study.

Further investigations should include a complete cardiovascular assessment in order to understand the possible effect of cardiac workload improvement on the peripheral fluid reabsorption. Finally, long term follow-up is also needed to establish how much of all the registered variations can be maintained along the time.

## Figures and Tables

**Table 1:** Euganean Hill thermal basin chemical–physical water properties.

Properties	Value
Temperature before arriving at the surface	80/90 °C
pH	7.1
Conductivity	7042 μS/cm
Fixed residue at 180°	5050 mg/L
Sodium (Na^+^)	1239 mg/L
Potassium (K^+^)	88 mg/L
Calcium (Ca^2+^)	3667 mg/L
Magnesium (Mg^2+^)	80 mg/L
Silica (SiO^2^)	51 mg/L
Ammonium (NH_4+_)	2.7 mg/L
Total iron	<0.05 mg/L
Sulfate (SO_42−)_	980 mg/L
Chlorides (Cl^−^)	2176 mg/L
Bicarbonate (HCO^3−^)	169 mg/L
Bromide (Br^−^)	13.6 mg/L
Iodide (I^−^)	0.82 mg/L
Hydrogen Sulfide (H_2_S)	1.67 mg/L

**Table 2:** BIA (bioimpedance analysis) parameter changes along the exercise program.

	Baseline (T0)	7 Days (T7)	14 Day (T14)	*p* Value (T0-T7)	*p* Value (T7-T14)	*p* Value (T0-T14)
**% ECW**	42.1 ± 5.8	41.8 ± 5.5	41.2 ± 5.5	0.001	0.03	0.001
**R (Ohm)**	455.4 ± 78.2	471. 0 ± 81.1	488.1 ± 83.6	0.001	0.05	0.0009
**Xc (Ohm)**	53.1 ± 11.9	54.2 ± 11.7	57.6 ± 14.7	0.2602	0.04	0.009
**PA °**	6.6 ±0.7	6.5 ± 0.8	6.6 ± 0.9	0.3761	0.2305	0.5610

% ECW= extracellular water rate; R = resistance; Xc = reactance; PA = phase angle.

## References

[1] Lurie F., Passman M., Meisner M., Dalsing M., Masuda E., Welch H., Bush R.L., Blebea J., Carpentier P.H., De Maeseneer M. (2020). The 2020 update of the CEAP classification system and reporting standards. J. Vasc. Surg. Venous Lymphat. Disord..

[2] Leu A.J., Leu H.J., Franzeck U.K., Bollinger A. (1995). Microvascular changes in chronic venous insufficiency-a review. Cardiovasc Surg..

[3] Bunke N., Brown K., Bergan J. (2009). Phlebolymphedema: Usually unrecognized, often poorly treated. Perspect Vasc. Surg. Endovasc. Ther..

[4] Khalil S.F., Mohktar M.S., Ibrahim F. (2014). The theory and fundamentals of bioimpedance analysis in clinical status monitoring and diagnosis of diseases. Sensors (Basel).

[5] Martinsen O.G., Grimnes S. (2011). Bioimpedance and Bioelectricity Basics.

[6] Mulasi U., Kuchnia A.J., Cole A.J., Earthman C.P. (2015). Bioimpedance at the bedside: Current applications, limitations, and opportunities [published correction appears in Nutr Clin Pract. 2015, 30:589]. Nutr. Clin. Pract..

[7] Sergi G., Bussolotto M., Perini P., Calliari I., Giantin V., Ceccon A., Scanferla F., Bressan M., Moschini G., Enzi G. (1994). Accuracy of bioelectrical impedance analysis in estimation of extracellular space in healthy subjects and in fluid retention states. Ann. Nutr. Metab..

[8] Sargent J.A., Gotch F.A. (1989). Principles and biophysics of dialysis. Replacement of Renal Function by Dialysis.

[9] Maffrin M.Y., Morel H. (2008). Body fluid volumes measurements by impedance: A review of bioimpedance spectroscopy (BIS) and bioimpedance analysis (BIA) methods. Med. Eng. Phys..

[10] Earthman C., Traughber D., Dobratz J., Howell W. (2007). Bioimpedance spectroscopy for clinical assessment of fluid distribution and body cell mass. Nutr. Clin. Pract..

[11] Matthie J.R., Withers P.O. (1998). Bioimpedance: 50 kHz parallel reactance and the prediction of body cell mass. Am. J. Clin. Nutr..

[12] Gaw R., Box R., Cornish B. (2011). Bioimpedance in the assessment of unilateral lymphedema of a limb: The optimal frequency. Lymphat. Res. Biol..

[13] Cavezzi A., Urso S.U., Paccasassi S., Mosti G., Campana F., Colucci R. (2020). Bioimpedance spectroscopy and volumetry in the immediate/short-term monitoring of intensive complex decongestive treatment of lymphedema. Phlebology.

[14] Mattiello R., Amaral M.A., Mundstock E., Ziegelmann P.K. (2020). Reference values for the phase angle of the electrical bioimpedance: Systematic review and meta-analysis involving more than 250,000 subjects. Clin. Nutr..

[15] Kumar S., Dutt A., Hemraj S., Bhat S., Manipadybhima B. (2012). Phase Angle Measurement in Healthy Human Subjects through Bio-Impedance Analysis. Iran J. Basic Med. Sci..

[16] Ward L.C., Bunce I.H., Cornish B.H., Mirolo B.R., Thomas B.J., Jones L.C. (1992). Multi-frequency bioelectrical impedance augments the diagnosis and management of lymphoedema in post-mastectomy patients. Eur. J. Clin. Investig..

[17] Choi Y.H., Seo K.S. (2014). Correlation among bioimpedance analysis, sonographic and circumferential measurement in assessment of breast cancer-related arm lymphedema. Lymphology.

[18] Coroneos C.J., Wong F.C., DeSnyder S.M., Shaitelman S.F., Schaverien M.V. (2019). Correlation of L-Dex Bioimpedance Spectroscopy with Limb Volume and Lymphatic Function in Lymphedema. Lymphat. Res. Biol..

[19] Bundred N.J., Stockton C., Keeley V., Riches K., Ashcroft L., Evans A., Skene A., Purushotham A., Bramley M., Hodgkiss T. (2015). Investigators of BEA/PLACE studies. Comparison of multi-frequency bioimpedance with perometry for the early detection and intervention of lymphoedema after axillary node clearance for breast cancer. Breast Cancer Res Treat..

[20] Cornish B.H., Chapman M., Hirst C., Mirolo B., Bunce I.H., Ward L.C., Thomas B.J. (2001). Early diagnosis of lymphedema using multiple frequency bioimpedance. Lymphology.

[21] Cho K.H., Han E.Y., Lee S.A., Park H., Lee C., Im S.H. (2020). Feasibility of Bioimpedance Analysis to Assess the Outcome of Complex Decongestive Therapy in Cancer Treatment-Related Lymphedema. Front Oncol..

[22] Dionne A., Goulet S., Leone M., Comtois A.S. (2018). Aquatic Exercise Training Outcomes on Functional Capacity, Quality of Life, and Lower Limb Lymphedema: Pilot Study. J. Altern. Complement. Med..

[23] Ergin G., Karadibak D., Sener H.O., Gurpinar B. (2017). Effects of Aqua-Lymphatic Therapy on Lower Extremity Lymphedema: A Randomized Controlled Study. Lymphat. Res. Biol..

[24] Yeung W., Semciw A.I. (2018). Aquatic Therapy for People with Lymphedema: A Systematic Review and Meta-analysis. Lymphat. Res. Biol..

[25] Iyer N.S., Cartmel B., Friedman L., Li F., Zhou Y., Ercolano E., Harrigan M., Gottlieb L., McCorkle R., Schwartz P.E. (2018). Lymphedema in ovarian cancer survivors: Assessing diagnostic methods and the effects of physical activity. Cancer.

[26] Fukushima T., Tsuji T., Sano Y., Miyata C., Kamisako M., Hohri H., Yoshimura C., Asakura M., Okitsu T., Muraoka K. (2017). Immediate effects of active exercise with compression therapy on lower-limb lymphedema. Support Care Cancer.

[27] Gianesini S., Tessari M., Bacciglieri P., Malagoni A.M., Menegatti E., Occhionorelli S., Basaglia N., Zamboni P. (2017). A specifically designed aquatic exercise protocol to reduce chronic lower limb edema. Phlebology.

[28] Musumeci A., Pranovi G., Masiero S. (2018). Patient education and rehabilitation after hip arthroplasty in an Italian spa center: A pilot study on its feasibility. Int. J. Biometeorol..

[29] Gianesini S., Raffetto J.D., Mosti G., Maietti E., Sibilla M.G., Zamboni P., Menegatti E. (2020). Volume control of the lower limb with graduated compression during different muscle pump activation conditions and the relation to limb circumference variation. J. Vasc. Surg. Venous Lymphat. Disord..

[30] Rabe E., Stucker M., Ottillinger B. (2010). Water displacement leg volumetry in clinical studies–A discussion of error sources. BMC Med. Res. Methodol..

[31] Perrin M., Guex J.J. (2000). Edema and leg volume: Methods of assessment. Angiology.

[32] Schober P., Boer C., Schwarte L.A. (2018). Correlation Coefficients: Appropriate Use and Interpretation. Anesth Analg..

[33] Caggiati A., Lattimer C., Kalodiki E., Oberto S., Bergamo G., Kontothanassis D. (2018). Underwater Sonography of Leg Veins. EJVES Short Rep..

[34] Masiero S., Vittadini F., Ferroni C., Bosco A., Serra R., Frigo A.C., Frizziero A. (2018). The role of thermal balneotherapy in the treatment of obese patient with knee osteoarthritis. Int. J. Biometeorol..

[35] Cozzi F., Carrara M., Sfriso P., Todesco S., Cima L. (2004). Anti-inflammatory effect of mud-bath applications on adjuvant arthritis in rats. Clin. Exp. Rheumatol..

[36] Bellometti S., Galzigna L. (1998). Serum levels of a prostaglandin and a leukotriene after thermal mud pack therapy. J. Investig. Med..

[37] Bellometti S., Galzigna L., Richelmi P., Gregotti C., Bertè F. (2002). Both serum receptors of tumor necrosis factor are influenced by mud pack treatment in osteoarthrotic patients. Int. J. Tissue React..

[38] Bacolla A., Giraudi G., Lorenzini P., Varacca G., Costa A. (1983). Observations in iodine exchange in thermal therapy with salsobromoiodic water. Panminerva Med..

[39] Ippolito E., De Luca S., Sommaruga S., Grassellino V., Nappi G. (2008). Experimental-clinical study on the effects of hydromassage with Thermae Oasis’s salsobromoiodine water in chronic venous stasis disease of the lower extremities. Minerva Cardioangiol..

[40] Kimura G. (2005). Pathogenesis of edema and its classification. Nihon Rinsho. Jpn. J. Clin. Med..

[41] Cavezzi A., Mosti G., Colucci R., Quinzi V., Bastiani L., Urso S.U. (2019). Compression with 23 mmHg or 35 mmHg stockings after saphenous catheter foam sclerotherapy and phlebectomy of varicose veins: A randomized controlled study. Phlebology.

[42] Genton L., Norman K., Spoerri A., Pichard C., Karsegard V.L., Herrmann F.R., Graf C.E. (2017). Bioimpedance-Derived Phase Angle and Mortality Among Older People. Rejuvenation Res..

[43] Aukland K. (1984). Distribution of body fluids: Local mechanisms guarding interstitial fluid volume. J. Physiol. (Paris).

[44] Lurie F. (2014). Physiology and Pathophysiology of the Venous System. PanVascular Med..

[45] Raju S., Varney E., Flowers W., Cruse G. (2016). Effect of External Positive and Negative Pressure on Venous Flow in an Experimental Model. Eur. J. Vasc. Endovasc. Surg..

[46] Sarin S., Scurr J.H., Coleridge Smith P.D. (1992). Mechanism of action of external compression on venous function. Br. J. Surg..

[47] Tisato V., Zauli G., Voltan R., Gianesini S., di Iasio M.G., Volpi I., Fiorentini G., Zamboni P., Secchiero P. (2012). Endothelial cells obtained from patients affected by chronic venous disease exhibit a pro-inflammatory phenotype. PLoS ONE.

[48] Tisato V., Zauli G., Gianesini S., Menegatti E., Brunelli L., Manfredini R., Zamboni P., Secchiero P. (2014). Modulation of circulating cytokine-chemokine profile in patients affected by chronic venous insufficiency undergoing surgical hemodynamic correction. J. Immunol. Res..

[49] Tessari M., Tisato V., Rimondi E., Zamboni P., Malagoni A.M. (2018). Effects of intermittent pneumatic compression treatment on clinical outcomes and biochemical markers in patients at low mobility with lower limb edema. J. Vasc. Surg. Venous Lymphat. Disord..

[50] Becker B.E. (2009). Aquatic therapy: Scientific foundations and clinical rehabilitation applications. PM R.

[51] Michalsen A., Lüdtke R., Bühring M., Spahn G., Langhorst J., Dobos G.J. (2003). Thermal hydrotherapy improves quality of life and hemodynamic function in patients with chronic heart failure. Am. Heart J..

